# MRE11 and EXO1 nucleases degrade reversed forks and elicit MUS81-dependent fork rescue in BRCA2-deficient cells

**DOI:** 10.1038/s41467-017-01180-5

**Published:** 2017-10-16

**Authors:** Delphine Lemaçon, Jessica Jackson, Annabel Quinet, Joshua R. Brickner, Shan Li, Stephanie Yazinski, Zhongsheng You, Grzegorz Ira, Lee Zou, Nima Mosammaparast, Alessandro Vindigni

**Affiliations:** 1 Edward A. Doisy Department of Biochemistry and Molecular Biology, Saint Louis University School of Medicine, St Louis, MO 63104 USA; 2 Department of Pathology and Immunology, Division of Laboratory and Genomic Medicine, Washington University School of Medicine, St Louis, MO 63110 USA; 3 Department of Cell Biology and Physiology, Washington University School of Medicine, Campus Box 8228, 660S. Euclid Ave., St Louis, MO 63110 USA; 4 Massachusetts General Hospital Cancer Center, Harvard Medical School, Boston, MA 02129 USA; 5 Department of Molecular and Human Genetics, Baylor College of Medicine, One Baylor Plaza, Houston, TX 77030 USA

## Abstract

The breast cancer susceptibility proteins BRCA1 and BRCA2 have emerged as key stabilizing factors for the maintenance of replication fork integrity following replication stress. In their absence, stalled replication forks are extensively degraded by the MRE11 nuclease, leading to chemotherapeutic sensitivity. Here we report that BRCA proteins prevent nucleolytic degradation by protecting replication forks that have undergone fork reversal upon drug treatment. The unprotected regressed arms of reversed forks are the entry point for MRE11 in BRCA-deficient cells. The CtIP protein initiates MRE11-dependent degradation, which is extended by the EXO1 nuclease. Next, we show that the initial limited resection of the regressed arms establishes the substrate for MUS81 in BRCA2-deficient cells. In turn, MUS81 cleavage of regressed forks with a ssDNA tail promotes POLD3-dependent fork rescue. We propose that targeting this pathway may represent a new strategy to modulate BRCA2-deficient cancer cell response to chemotherapeutics that cause fork degradation.

## Abstract (web summary)

BRCA proteins have emerged as key stabilizing factors for the maintenance of replication forks following replication stress. Here the authors describe how reversed replication forks are degraded in the absence of BRCA2, and a MUS81 and POLD3-dependent mechanism of rescue following the withdrawal of genotoxic agent.

## Introduction

Germline mutations in the Breast Cancer Susceptibility genes *BRCA1/BRCA2* account for the vast majority of familial breast cancer cases^1–4^. Aside from their well-established roles in homologous recombination (HR), BRCA proteins are emerging as key factors required for the maintenance of replication fork stability following replication stress induction^5–8^. In their absence, replication forks are extensively degraded by the MRE11 nuclease. MRE11-dependent degradation of replication forks observed in the absence of BRCA proteins leads to long stretches of ssDNA (>4–5 kb) and is emerging as one of the leading causes of the sensitivity to therapies that target DNA or that inhibit specific repair pathways such as PARP inhibitors^5^. The mechanism leading to this extensive fork degradation phenotype in the absence of BRCA1 or BRCA2 remains unclear. For example, the exact structure(s) of the replication intermediates targeted by nucleases in BRCA-deficient cells is unknown. Moreover, MRE11 has limited nucleolytic activity^9^ and is unlikely to be the only nuclease responsible for degrading several kb of DNA in BRCA-deficient cells. Finally, the fate of the extensively resected forks upon drug removal has never been investigated in detail, even though it is tightly linked to the increased chromosomal aberrations and DNA damage sensitivity of BRCA-deficient cells.

Replication fork reversal is a key protective mechanism that allows replication forks to reverse their course when they encounter DNA lesions^10–14^. Interestingly, the same HR factors controlling MRE11 nuclease activity and ssDNA accumulation are also emerging as crucial players involved in fork remodeling^14–16^. In particular, the central recombinase RAD51 is essential for fork reversal upon chemotherapeutic treatment^14^. By analogy with its bacterial homologue RecA, RAD51 may be recruited to ssDNA stretches formed at replication fork junctions and promote the initial step of fork reversal by invading the complementary strand. In this context, HR proteins may also be required to stabilize forks in their reversed state by protecting the double-stranded end of the regressed arm from nucleolytic degradation.

In this study, we combine electron microscopy (EM) with genome-wide single-molecule DNA fiber approaches to define the mechanism by which the BRCA proteins protect replication forks from nucleolytic degradation following replication stress induction. We show that the main function of BRCA proteins in this context is to protect the regressed arms of replication forks that have reversed upon drug treatment from nucleolytic degradation. In their absence, CtIP initiates the MRE11-dependent degradation of the unprotected regressed arms and EXO1 contributes to extend fork degradation. Next, we investigate how cells cope with these extensively resected forks upon drug removal. In particular, we find that MUS81 cleavage rescues the resected forks in BRCA2-, but not BRCA1-deficient cells through a break-induced replication (BIR)-like mechanism mediated by POLD3-dependent DNA synthesis. Our findings revisit the functions of central HR factors in DNA replication and are crucial to understanding how targeting BIR-dependent pathways can modulate current chemotherapeutic modalities.

### EXO1 contributes to fork resection in BRCA-deficient cells

**Fig. 1:**
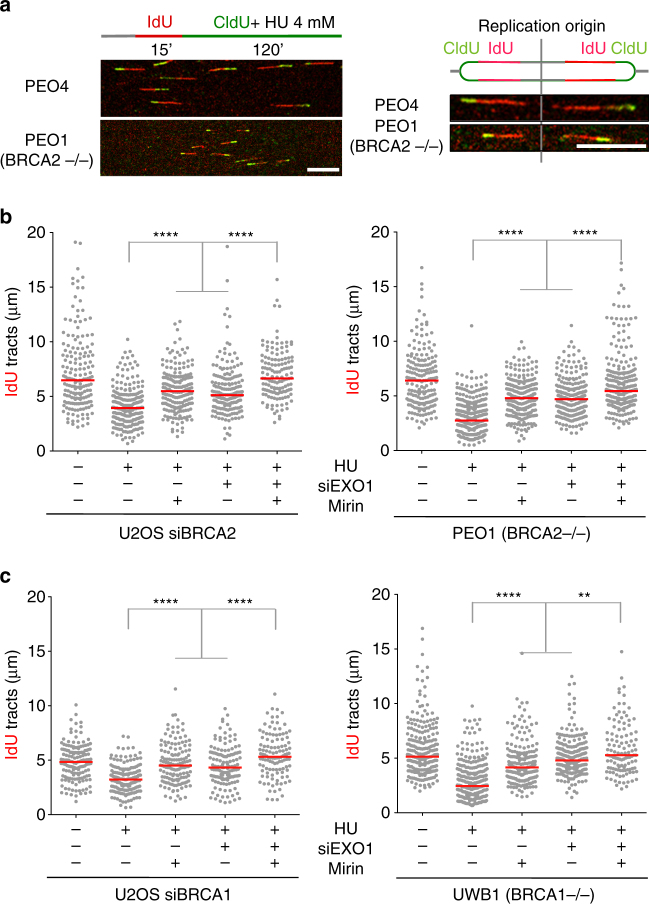
MRE11 and EXO1 mediate extended nascent strand degradation in HU-treated BRCA1- and BRCA2-deficient cancer cells. **a** Schematic of the single-molecule DNA fiber tract analysis and representative DNA fiber images of PEO4 and PEO1 cells treated with HU (4 mM) for 120 min. IdU, red; CldU, green. Scale bar, 50 μm. **b** Size distribution of IdU tract length in BRCA2-deficient U2OS (left) and PEO1 (right) cells in the presence and absence of HU. Cells were transfected with control siRNA or *EXO1* siRNA before IdU and CldU labeling. Mirin (50 μM) was added concomitantly with HU treatment, as indicated. Out of 3 repeats; *n* ≥ 250 tracts scored for each data set. Bars represent the median. Statistics: Mann–Whitney; ****P < 0.0001. **c** Size distribution of IdU tract length in BRCA1-deficient U2OS (left) and UWB1 (right) cells in the presence and absence of HU. Cells were transfected with control siRNA or *EXO1* siRNA before IdU and CldU labeling. Mirin (50 μM) was added concomitantly with HU treatment, as indicated. Out of 2 repeats; *n* ≥ 250 tracts scored for each data set. Bars represent the median. Statistics: Mann–Whitney; **P < 0.01; ****P < 0.0001

Two distinct pathways act redundantly to mediate processive double-strand break (DSB) resection downstream from the MRE11-RAD50-NBS1 (MRN) and CtIP factors in eukaryotic cells: one requires DNA2 and the other EXO1^17–21^. We sought to investigate whether DNA2 and EXO1 also contribute to the extended fork degradation phenotype of BRCA1- or BRCA2-deficient cells following genotoxic stress induction. We knocked down EXO1 or DNA2 in different cell lines, including the *BRCA2*-mutant ovarian cancer cells PEO1 (and the isogenic PEO4 cells, which contain a second point mutation restoring BRCA2 function), the Fanconi anemia *BRCA2*-mutant line EUFA423 (and its derivative expressing wild-type BRCA2), the *BRCA1* mutant ovarian cancer cell line UWB1.289 (and its complemented derivative expressing wild-type BRCA1), plus the human osteoscarcoma U2OS cells, which were siRNA-depleted for *BRCA1* or *BRCA2*. Nucleolytic resection following replication fork stalling was monitored by pulse-labeling cells with the first thymidine analog IdU (red label) followed by treatment with hydroxyurea (HU) and concomitant labeling with the second thymidine analog, CldU (green label) (Fig. 1a). Shortening of the first tract was measured as a readout of degradation only on forks characterized by contiguous IdU-CldU signals (and not on forks that have only the IdU label) to ensure that the shortening phenotype is indeed due to nucleolytic resection of stalled replication forks and not to premature termination events^22^. Upon HU treatment, BRCA1- and BRCA2-deficient cells showed a marked reduction (30–50% corresponding to > 5 kb of DNA) in the IdU tract length (Fig. 1b, c and Supplementary Fig. 1a). Moreover, MRE11 inhibition or knockdown partially rescued fork degradation consistent with the previous data^5–7^ (Fig. 1b, c and Supplementary Fig. 1). Analogous results were obtained using an alternative labeling scheme, where HU was added after thymidine labeling, suggesting that the results were not affected by the particular labeling scheme used in our work (Supplementary Fig. 1b). EXO1 knockdown by two different siRNAs yielded the same fork protection phenotype observed with MRE11 inhibition, indicating that EXO1 also contributes to fork degradation in BRCA1- and BRCA2-deficient cells upon HU treatment (Fig. 1 and Supplementary Figs. 1a–e and 2a, b). Interestingly, combined ablation of MRE11 and EXO1 activities further rescued the fork degradation phenotype of BRCA-deficient cells, suggesting that the two nucleases may be able to act independently on the stalled replication intermediates. The same results were obtained by treating BRCA2-deficient cells with DNA damaging agents such as cisplatin or UV-C, supporting the notion that different genotoxic agents trigger a similar fork resection mechanism whereby MRE11 and EXO1 extensively degrade replication forks in the absence of key HR factors (Supplementary Fig. 3a, b). Conversely, DNA2 knockdown did not restore fork protection (Supplementary Fig. 2c–g), in agreement with previous findings^23^. The same results were recapitulated by treating BRCA2-deficient cells with the NSC-105808 DNA2 inhibitor^24^ (Supplementary Fig. 2e). Taken together, these results suggest that human EXO1, but not DNA2, contributes to replication fork degradation in BRCA1- and BRCA2-deficient cells.

### MRE11 and EXO1 target reversed forks in BRCA-deficient cells

**Fig. 2:**
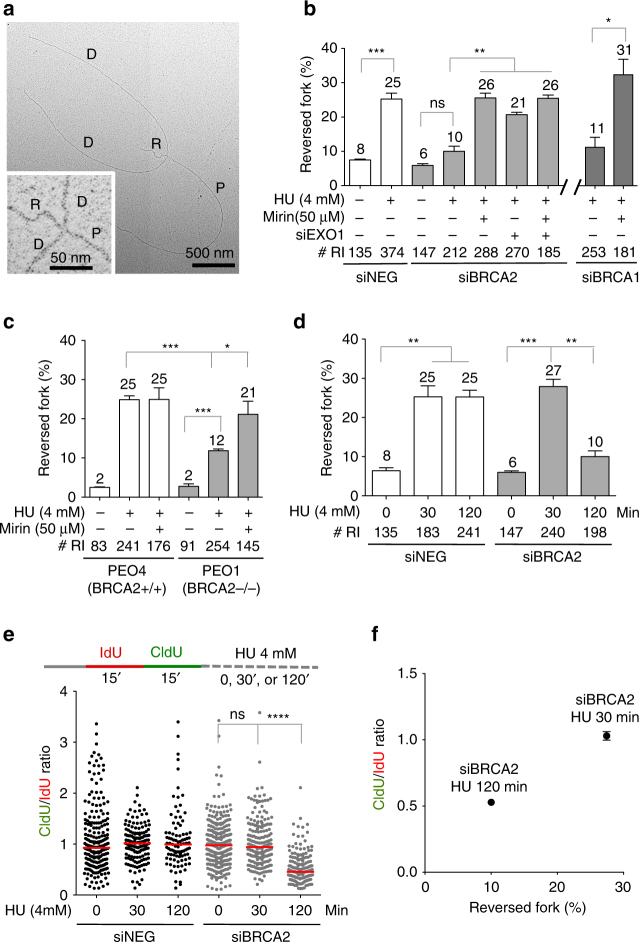
BRCA1 and BRCA2 protect reversed replication forks from MRE11- and EXO1-dependent degradation following HU treatment. **a** Representative electron micrograph of a reversed fork observed on genomic DNA upon HU treatment. Inset, magnified four-way junction at the reversed replication fork. D daugher strand, P parental strand, R reversed arm. **b** Frequency of fork reversal in BRCA1- and BRCA2-deficient U2OS cells treated with 4 mM HU for 5 h. Cells were transfected with control siRNA (siNEG) or *EXO1* siRNA. Mirin (50 μM) was added concomitantly with HU, as indicated. The percentage values are indicated on the top of the bar. “# RI” indicates the number of analyzed replication intermediates. Mean shown, *n* = 3. Errors, S.E.M. Statistics: unpaired *t* test; *P < 0.05; **P < 0.01; ***P < 0.001. **c** Frequency of fork reversal in BRCA2-proficient PEO4 and BRCA2-deficient PEO1 cells treated with 4 mM HU for 5 h. Mirin (50 μM) was added concomitantly with HU, as indicated. The percentage values are indicated on the top of the bar. “# RI” indicates the number of analyzed replication intermediates. Mean shown, *n* = 3. Errors, S.E.M. Statistics: unpaired *t* test; *P < 0.05; ***P < 0.001. **d** Frequency of fork reversal in BRCA2-deficient U2OS cells treated with 4 mM HU for 0, 30, or 120 min. The percentage values are indicated on the top of the bar. “# RI” indicates the number of analyzed replication intermediates. Mean shown, *n* = 3. Errors, S.E.M. Statistics: unpaired *t* test; **P < 0.01; ***P < 0.001. **e** CldU/IdU tract ratio in BRCA2-deficient U2OS cells treated with 4 mM HU for 0, 30, or 120 min. Out of 3 repeats; *n* ≥ 250 tracts scored for each data set. Bars represent the median. Statistics: Mann–Whitney; ****P < 0.0001. **f** Plot the percentages of reversed forks as a function of mean values of the CldU/IdU ratios measured after treating BRCA2-deficient U2OS cells with 4 mM HU for 30 or 120 min. Errors, S.E.M.

Next, we sought to investigate the actual structure(s) of the replication intermediates targeted by MRE11 and EXO1 in BRCA1- or BRCA2-deficient cells. We visualized the fine architecture of the replication intermediates using a combination of in vivo psoralen cross-linking and EM^25^ (Fig. 2a). Our analysis showed a substantial fraction of reversed replication forks (~25% of molecules analyzed) in control U2OS cells treated with 4 mM HU, consistent with previous data^14, 26^ (Fig. 2b). BRCA1- or BRCA2-knockdown resulted in a significantly lower frequency of fork reversal events (~10 and 11%, respectively) compared to HU-treated control cells, suggesting that BRCA proteins are required either to promote fork reversal or to prevent nucleolytic processing of forks that have already reversed following HU treatment. To distinguish between these two possibilities, we repeated the EM analysis while inhibiting MRE11 activity or knocking down EXO1 in BRCA-deficient cells. We found that ablation of either nuclease rescues the frequency of reversed forks to levels observed in control cells, suggesting that BRCA proteins protect forks that have already undergone fork reversal from nucleolytic degradation. Similar results were obtained by inhibiting MRE11 in BRCA2-mutant PEO1 cells (Fig. 2c). Time-course experiments performed by treating BRCA2-deficient U2OS cells with HU for 0, 30, or 120 min indicated that fork reversal precedes fork degradation, as predicted by our model (Fig. 2d–f). Collectively, these results suggest that BRCA proteins protect reversed forks from nucleolytic degradation and that the unprotected reversed forks are the entry point for MRE11 and EXO1 in BRCA1- and BRCA2-deficient cells.

### CtIP initiates MRE11-dependent resection of reversed forks

**Fig. 3:**
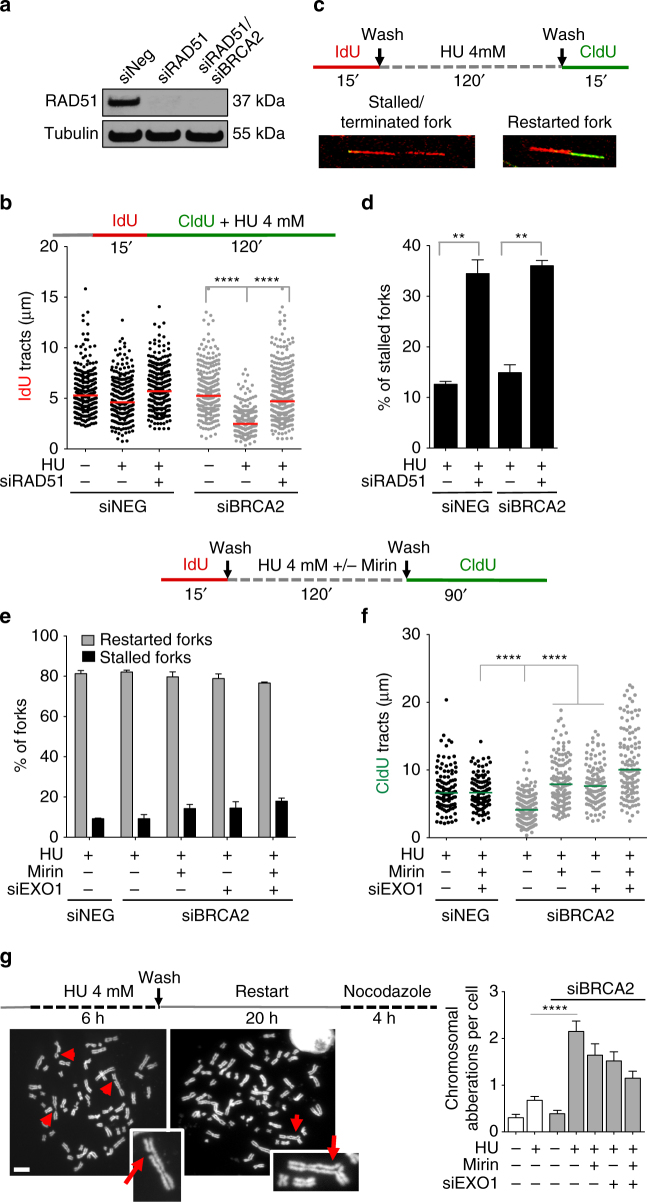
Resected forks are able to restart in BRCA2-deficient cells and lead to increased chromosomal aberrations. **a** Expression of RAD51 after siRNA knockdown in U2OS cells. **b** Size distribution of IdU tract length in BRCA2-deficient and -proficient U2OS cells in the presence and absence of HU. Cells were transfected with control siRNA (siNEG), *RAD51* siRNA, or *BRCA2* siRNA before IdU and CldU labeling. Out of 2 repeats; *n* ≥ 250 tracts scored for each data set. Bars represent the median. Statistics: Mann–Whitney; ****P < 0.0001. **c** Schematic of the single-molecule DNA fiber tract analysis for the fork restart experiments. Red-green contiguous tracts (restarting forks). Red only tracts (stalled/terminated forks). **d** Quantification of restarting forks in BRCA2-deficient and -proficient U2OS cells with or without *RAD51* siRNA knockdown. Out of 2 repeats, the percentage is established on at least 250 tracts scored for each data set. Mean shown. Errors, S.E.M. Statistics: unpaired *t* test; **P < 0.01. **e** Quantification of restarting forks in BRCA2-deficient and -proficient U2OS cells with or without *EXO1* siRNA knockdown. Mirin (50 μM) was added concomitantly with HU treatment, as indicated. Out of 2 repeats, the percentage is established on at least 250 tracts scored for each data set. Mean shown. Errors, S.E.M. **f** Size distribution of CldU tract length in BRCA2-deficient and -proficient U2OS cells in the presence of HU. Cells were transfected with control siRNA (siNEG), *EXO1* siRNA, or *BRCA2* siRNA before IdU and CldU labeling. Mirin (50 μM) was added concomitantly with HU treatment, as indicated. Out of 2 repeats; *n* ≥ 250 tracts scored for each data set. Bars represent the median. Statistics: Mann–Whitney; ****P < 0.0001. **g** Chromosomal aberrations in BRCA2-deficient and -proficient U2OS cells in the presence of HU. Left, representative images of metaphase spreads in the presence of HU. Scale bar, 25 μm. Sketch above the images delineates experimental design. Right, numbers of chromosomal aberrations per metaphase are plotted. At least 50 metaphases counted in each experiments. Mean shown, *n* = 3.. Errors, S.E.M. Statistics: unpaired *t* test: ****P < 0.0001

The regressed arm of a reversed replication fork resembles by all means a one-ended DSB. Previous biochemical studies showed that the 5′–3′ endonuclease activity of MRE11 initiates the resection process and that CtIP is required to promote MRE11 endonuclease activity at the 5′ strand^27^. Resection is then continued by the 5′–3′ exonuclease activity of EXO1^19, 28, 29^. As an alternative approach to confirm that MRE11-dependent resection starts from the regressed arm of reversed replication forks, we repeated the DNA fiber experiments with CtIP knockdown cells and found that CtIP loss prevents fork degradation in BRCA2-deficient cells (Supplementary Fig. 3c, d). These data suggest that the same pathway that initiates DSB resection in the context of HR is required to process the open dsDNA end of the regressed arm. To further validate this conclusion, we reasoned that degradation should not take place in a genetic background that prevents reversed fork formation—i.e., RAD51 knockdown^14^. Indeed, loss of RAD51 suppressed fork degradation in BRCA2-depleted U2OS cells, confirming that RAD51 acts upstream of BRCA2 to promote reversed fork formation (Fig. 3a, b). In addition, RAD51 depletion severely compromised fork restart, suggesting that fork remodeling is an essential requirement for efficient resumption of DNA synthesis upon HU removal (Figs. 3c, d).

### Resection does not affect fork restart upon BRCA2 loss

The extended nucleolytic degradation of stalled replication intermediates in BRCA1- and BRCA2-deficient cells leads to increased chromosomal aberrations and genome instability^5, 6^. However, the molecular mechanism linking fork degradation with chromosomal instability remains elusive. Thereby, we set out to investigate how cells cope with these extensively degraded forks focusing on BRCA2-deficient cells. Fork degradation associated with BRCA2 loss did not significantly affect fork restart (Fig. 3e), in agreement with previous findings^5, 6^. However, by increasing the timing of CldU labeling from 15 to 90 min, we found that thymidine incorporation after HU removal was reduced in BRCA2-deficient compared to BRCA2-proficient cells (Fig. 3f). This reduction in the CldU tract length suggests that BRCA2 loss, though not affecting the number of restarting forks, either impairs fork progression after restart or causes a delayed restart of the resected forks. Ablation of MRE11 and/or EXO1 activity rescued CldU tract length, suggesting that the extended nascent strand degradation associated with BRCA2 loss is the leading cause of the observed defect in fork progression or timing of restart (Fig. 3f). Moreover, BRCA2 loss led to increased chromosomal aberrations when cells were challenged with HU, and this effect was again partially rescued by ablation of MRE11 and/or EXO1 activity, supporting a link between fork degradation, defects in fork progression or restart upon drug removal and increased chromosomal aberrations (Fig. 3g).

### MUS81 cleaves partially resected reversed forks

**Fig. 4:**
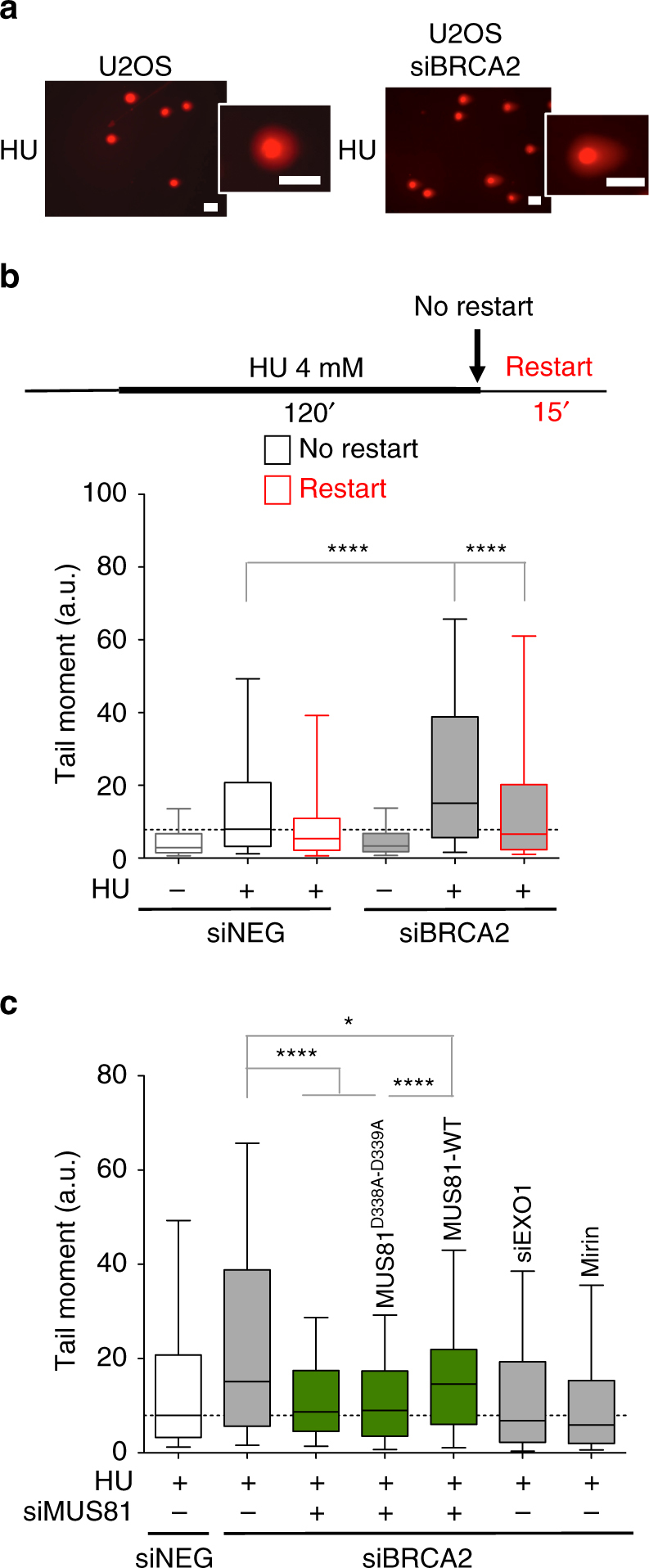
MUS81 cleavage leads to transient DSB accumulation in BRCA2-deficient cells. **a** Representative comet images of BRCA2-proficient and -deficient cells following treatment with 4 mM HU for 120 min. Scale bar, 50 μm. **b** Neutral Comet assay monitoring DSB formation in BRCA2-deficient and -proficient U2OS cells following HU treatment for 120 min (no restart) and 15 min after HU removal (restart). Cells were transfected with control siRNA (siNEG) or *BRCA2* siRNA. Out of 3 repeats; *n* ≥ 200 comets scored for each data set. Whiskers the 10th and 90th percentiles. ****P < 0.0001 (Mann–Whitney test). **c** Neutral Comet assay monitoring DSB formation upon MUS81 depletion or complementation wild-type (MUS81-WT) or catalytically dead (MUS81^D338A-D339A^) MUS81. Cells were transfected with control siRNA (siNEG), *BRCA2* siRNA, *MUS81* siRNA, or *EXO1* siRNA. MUS81-depleted cells were complemented with wild-type (MUS81-WT) or catalytically dead (MUS81^D338A-D339A^) MUS81, when indicated. Mirin (50 μM) was added concomitantly with HU treatment, as indicated. Out of 3 repeats; *n* ≥ 200 comets scored for each data set Whiskers the 10th and 90th percentiles. ****P < 0.0001, *P < 0.05 (Mann–Whitney test)

We next examined the mechanism that rescues resected forks upon drug removal. Using neutral comet assays we found that BRCA2 loss leads to DSB accumulation upon HU treatment (Figs. 4a, b). However, the frequency of DSBs decreased to control levels 15 min after HU removal, suggesting that these DSBs are quickly repaired after fork restart. Interestingly, MUS81 depletion prevented DSB accumulation in *BRCA2*-deficient cells (Fig. 4c). A short-interfering RNA (siRNA)-resistant *MUS81* cDNA, but not a catalytically inactive version (*MUS81(D338A/D339A)*), restored DSB accumulation in *MUS81* siRNA-depleted cells (Fig. 4c and Supplementary Fig. 3f). Therefore, we propose that MUS81 endonuclease activity is required to cleave partially resected forks, leading to transient DSB accumulation in the absence of BRCA2. Our observation that MUS81 loss did not significantly affect fork resection supports the idea that the extended fork degradation phenotype was not due to the resection of the DSBs created by MUS81 cleavage (Supplementary Fig. 3g). Moreover, the notion that MUS81 acts downstream of MRE11 and EXO1 was supported by the observation that the DSB accumulation of BRCA2-deficent cells was significantly reduced in a genetic background that prevents fork resection—i.e., loss of MRE11, EXO1, or CtIP activity (Fig. 4c and Supplementary Fig. 3e).

**Fig. 5:**
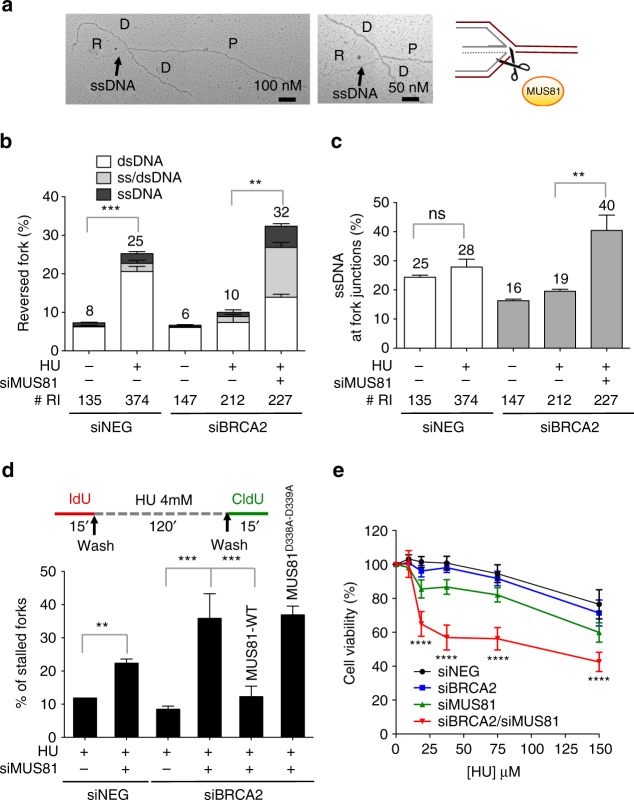
MUS81 cleaves partially resected regressed forks with a ssDNA tail to promote fork rescue in BRCA2-deficient cells. D daughter strand, P parental strand, R reversed arm. **a** Left, representative electron micrograph of a reversed fork with a single-stranded regressed arm. Center, magnified four-way junction at the reversed replication fork with a single-stranded regressed arm. The black arrow points to the ssDNA region on the regressed arm. Right, schematic model of the substrate cleaved by MUS81. **b** Frequency of fork reversal and ssDNA composition of the reversed arms in *BRCA2*-deficient U2OS cells treated with 4 mM HU for 5 h. Cells were transfected with control siRNA (siNEG) or *MUS81* siRNA. The percentage values are indicated on the top of the bar. “# RI” indicates the number of analyzed replication intermediates. Mean shown, *n* = 3. Errors, S.E.M. Statistics: unpaired *t* test; **P < 0.01; ***P < 0.001. **c** Percentage of forks with ssDNA at the fork junction in BRCA2-deficient U2OS cells treated with 4 mM HU for 5 h. Cells were transfected with control siRNA (siNEG) or *MUS81* siRNA. The percentage values are indicated on the top of the bar. “# RI” indicates the number of analyzed replication intermediates. Mean shown, *n* = 3. Errors, S.E.M. Statistics: unpaired *t* test; **P < 0.01. **d** Quantification of restarting forks in BRCA2-deficient and -proficient U2OS cells with or without *MUS81* siRNA knockdown. MUS81-depleted cells were complemented with wild-type (MUS81-WT) or catalytically dead (MUS81^D338A-D339A^) MUS81, when indicated. Out of 3 repeats, the percentage is established on at least 250 tracts scored for each data set. Mean shown. Errors, S.E.M. Statistics: unpaired *t* test; **P < 0.01; ***P < 0.001 **e** Cell viability assays 72 h upon treatment with the indicated doses of HU. U2OS cells were transfected with control siRNA (siNEG), *BRCA2* siRNA, or *MUS81* siRNA. Mean shown, *n* = 6. Errors, S.E.M. Statistics: two-way ANOVA, ***P > 0.001; ****P > 0.0001 (differences between *BRCA2* siRNA and *BRCA2/MUS81* siRNA)

To define the exact substrate cleaved by MUS81 in BRCA2-deficient cells, we inspected the structure of the replication intermediates that accumulate in BRCA2-deficient cells upon MUS81 depletion. Our EM analysis revealed that MUS81 loss causes a dramatic increase in reversed fork frequency in BRCA2-deficient cells (Fig. 5a, b). Next, we evaluated the ssDNA composition of the regressed arms by detecting local differences in filament thickness. MUS81 depletion led to a significant increase in the percentage of regressed forks that are partially or entirely single-stranded, strongly suggesting that MUS81 cleaves partially resected regressed forks with a ssDNA tail (Fig. 5b). Interestingly, MUS81 depletion also led to a significant increase in the percentage of daughter strands with ssDNA at the fork junction, suggesting that in the absence of MUS81 cleavage asymmetric fork resection continues beyond the length of the regressed arm, leading to partially resected 3-way junction structures (Fig. 5c). Of note, the small fraction of HU-induced DSBs of BRCA2-proficient cells is also rescued by the loss of MUS81, but not of MRE11 or EXO1 (Supplementary Fig. 3h). This observation is consistent with the previous biochemical data showing that MUS81 can also cleave intact forks, although with much lower efficiency compared to flap-fork substrates^30^. Taken together, these results suggest that the initial resection of the regressed arms leads to the formation of reversed forks with a ssDNA flap, which are cleaved by MUS81.

### MUS81 cleavage promotes POLD3-dependent DNA synthesis

To test whether MUS81-dependent cleavage of resected regressed forks is required for fork restart, we quantified the percentage of stalled forks in MUS81 and BRCA2 co-depleted cells (Fig. 5d). MUS81 loss slightly increased fork stalling in BRCA2-proficient cells, in agreement with previous findings^31, 32^. This effect was significantly more dramatic in the absence of BRCA2 and was confirmed using two different MUS81 siRNAs (Fig. 5d and Supplementary Fig. 4a, b). Genetic knockdown–rescue experiments confirmed that complementation in MUS81-depleted U2OS cells with siRNA-resistant wild-type MUS81, but not with the catalytically inactive mutant, abrogated the effect of MUS81 depletion on replication fork stalling upon HU treatment (Fig. 5d).

**Fig. 6:**
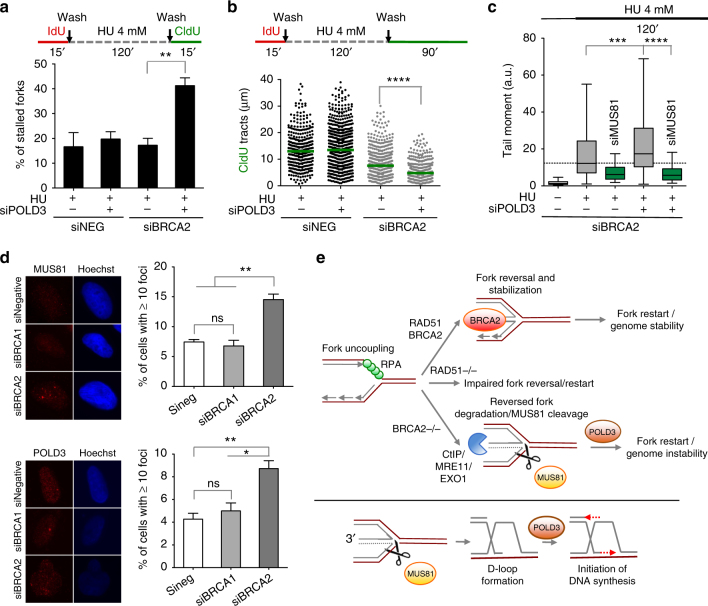
POLD3 is required to restart resected forks in BRCA2-deficient cells. **a** Quantification of restarting forks in BRCA2-deficient and -proficient U2OS cells with or without *POLD3* siRNA knockdown. Out of 2 repeats, the percentage is established on at least 250 tracts scored for each data set. Mean shown. Errors, S.E.M. Statistics: unpaired *t* test; **P < 0.01. **b** Size distribution of CldU tract length in U2OS cells transfected with control siRNA (siNEG), *POLD3* siRNA, or *BRCA2* siRNA before IdU and CldU labeling. Out of 3 repeats; *n* ≥ 150 tracts scored for each data set. Bars represent the median. Statistics: Mann–Whitney; ****P < 0.0001. **c** Neutral Comet assay monitoring DSB formation upon POLD3 depletion in BRCA2-deficient cells. Cells were transfected with control siRNA (siNEG), *BRCA2* siRNA, *MUS81* siRNA, or *POLD3* siRNA. Out of 2 repeats; *n* ≥ 200 comets scored for each data set. Whiskers the 10th and 90th percentiles. ***P < 0.001: ****P < 0.0001 (Mann–Whitney test). **d** Left, representative images of MUS81 and POLD3 foci observed upon HU treatment. Scale bar, 10 μm. U2OS cells were transfected with control *siRNA* (siNEG), *BRCA1* siRNA, or *BRCA2* siRNA and processed for immunofluorescence. Right, quantitation of MUS81 and POLD3 foci. Out of 3 repeats; *n* ≥ 150 cells scored for each data set. Mean shown. Errors, S.E.M. Statistics: unpaired *t* test; *P < 0.05; **P < 0.01. **e** Top, roles of BRCA2, CtIP/MRE11/EXO1 and MUS81/POLD3 in reversed fork protection, degradation and cleavage/restart, respectively. Genotoxic agents lead to fork uncoupling and reversal. Reversed forks are stabilized by BRCA2, allowing accurate fork restart and genome stability. BRCA1 shares a similar function in reversed fork stabilization. RAD51 loss prevents fork reversal and compromises fork restart and cell viability. BRCA2 loss leads to CtIP/MRE11/EXO1-mediated degradation of the reversed replication forks. MUS81 cleaves the partially resected reversed forks with a ssDNA flap to grant POLD3-dependent fork restart and cell survival. Bottom, the MUS81 endonuclease cleaves the resected reversed forks with a ssDNA flap and lead to the subsequent formation of a D-loop structure that would in turn initiate POLD3-dependent DNA synthesis

MUS81 has two partners in human cells, EME1 and EME2, and the two proteins are thought to interact with MUS81 at different stages of the cell cycle. Genetic knockdown experiments showed that EME2, but not EME1, is involved in the same fork restart pathway, suggesting that the function of the MUS81-EME2 complex is restricted to the S-phase (Supplementary Fig. 4c, d), in agreement with previous findings^32^. MUS81 cleavage was previously shown to promote POLD3-dependent DNA synthesis at common fragile sites^33^ and telomeric^34^ loci in human cells. POLD3 is one of the accessory subunits of the replicative polymerase POL δ and was recently shown to be required for BIR, a specialized HR pathway to repair DSBs at stalled forks^35, 36^. Similar to MUS81 depletion, POLD3 loss increased fork stalling in BRCA2-deficient cells (Fig. 6a and Supplementary Fig. 4e). Moreover, it led to a severe defect in fork progression, suggesting that POLD3-dependent DNA synthesis is required to restart the MUS81-cleaved resected forks (Fig. 6b). Interestingly, POLD3/BRCA2 double depletion caused a further increase in DSB accumulation compared to depletion of BRCA2 alone and these DSBs were again rescued by MUS81 depletion confirming that POLD3 acts downstream of MUS81 in the pathway (Fig. 6c). The notion that BRCA2-deficient cells rely on the MUS81-dependent pathway to resume DNA synthesis is supported by our observation that cell viability is reduced in MUS81/BRCA2 co-depleted cells relative to BRCA2-depleted cells following HU treatment (Fig. 5e). Conversely, PARP inhibitor sensitivity was not significantly affected by MUS81 depletion consistent with the notion that PARP inhibitors, differently from HU, prevent reversed fork accumulation^10, 13^ and thereby do not require a MUS81-dependent pathway of fork rescue (Supplementary Fig. 4f). Collectively, these results suggest that BRCA2-deficent cells rely on a MUS81/POLD3-dependent mechanism to rescue resected replication forks following treatment with genotoxic agents that induce replication fork reversal and degradation in a BRCA2-deficient background.

### MUS81 does not rescue forks in BRCA1-deficient cells

Our data suggest that BRCA1 shares a function similar to BRCA2 in reversed fork protection (Fig. 1). However, the MUS81 pathway is not required to rescue forks in BRCA1-deficient cells (Supplementary Fig. 4g, h), suggesting that different pathways mediate the restart of the resected forks depending on the particular genetic background. These findings are in agreement with recent studies, suggesting that MUS81 depletion differentially affects chemotherapeutic sensitivity in BRCA2- versus BRCA1-deficient cells (Alan D’Andrea personal communication) and with our immunofluorescence experiments showing that MUS81 and POLD3 foci accumulate specifically in BRCA2-, but not in BRCA1-deficient cells (Fig. 6d).

## Discussion

This work defines the mechanism that leads to the extensive fork degradation phenotype observed in BRCA2-deficient cells and provides novel insights into the molecular steps that rescue the resected forks upon drug removal. We propose a model whereby BRCA2 protects the regressed arms of replication forks that have reversed upon drug treatment from nucleolytic degradation. In its absence, the double-stranded ends formed by fork reversal are targeted by the CtIP, MRE11, and EXO1 nucleases to initiate the degradation of the stalled replication intermediates (Fig. 6e). Recent data suggest that RAD51 promotes reversed fork formation^14^ and is enriched on nascent DNA independently of BRCA2^5^. We propose that RAD51 has two distinct functions during replication stress: a BRCA2-independent function in promoting the initial step of reversed fork formation and a BRCA2-dependent function, whereby BRCA proteins protect the already formed reversed forks from nucleolytic degradation by stabilizing the RAD51 filament on the regressed arm. In BRCA2-deficient cells, this second function is lost, leading to the nascent strand degradation phenotype observed with BRCA2 mutants unable to stabilize RAD51 on ssDNA^6^ or with RAD51 mutants that destabilize the RAD51 nucleofilament^37, 38^. Because of the severe defect in fork restart associated with RAD51 depletion are reported here, we also suggest that fork remodeling is a central mechanism of replication stress response following prolonged drug treatment. Collectively, these findings shed light on the long-debated function of central HR factors in fork remodeling and go beyond the oversimplified concept that HR factors are simply required for DSB repair during replication stress. Our findings also reveal that MUS81-dependent cleavage of the resected forks is required for fork restart in BRCA2-deficient cells. The discovery that replication stalling induces RAD51 foci formation in a MUS81-independent manner is consistent with the idea that MUS81 is required to cleave forks that have already undergone RAD51-dependent fork remodeling^31^. We propose that MUS81 acts downstream of MRE11- and EXO1-mediated degradation. On the basis of our findings that CtIP is required to promote fork resection and MRE11 endonuclease activity at the 5′ strand, we also propose that the initial degradation of the regressed arms generates a reversed fork with a 3′-ssDNA tail that is then cleaved by MUS81 to mediate fork restart. This notion is consistent with the in vitro data showing that MUS81 efficiently cleaves 3′ flaps, Y-shaped structures and nicked Holliday junctions (HJ), but has negligible activity toward intact HJs (resembling an intact reversed fork substrate)^30, 39, 40^. In the absence of MUS81 cleavage, the nucleolytic degradation might quickly proceed to degrade nascent strands behind the junction finally leading to the extensively resected forks observed by DNA fiber (Supplementary Fig. 4i). Recent studies suggest that completion of DNA replication at common fragile sites^33^ and telomeric^34^ loci occurs via a specialized form of DNA repair originally characterized in yeast and termed BIR, whereby MUS81 cleavage of stalled replication forks produces a migrating bubble that drives POLD3-dependent DNA synthesis^41, 42, 43^. We propose that a similar mechanism is responsible to rescue partially resected regressed forks in BRCA2-deficient cells. We speculate that the MUS81/POLD3-dependent pathway used to rescue resected forks in BRCA2-deficient cells might represent a novel anticancer drug target specific for BRCA2-defective tumors to be used in combination with chemotherapeutics that cause replication fork reversal and degradation.

## Methods

### Cell lines and culture conditions

Cell lines: the human osteosarcoma U2OS cells (American Type Culture Collection), *BRCA2*-mutant ovarian cancer cells PEO1 (and the isogenic PEO4 cells, which contain a second point mutation restoring BRCA2 function) (provided by Dr. Lee Zou, Harvard Medical School)^44^, *Fanconi anemia BRCA2*-mutant line EUFA423 (and its derivative expressing wild-type BRCA2) (provided by Dr. Douglas Bishop, University of Chicago)^45^, *BRCA1* mutant ovarian cancer cell line UWB1.289 (and its complemented derivative expressing wild-type BRCA1) (provided by Dr. Lee Zou, Harvard Medical School)^46^. U2OS and EUFA 423 F/HAB2^45^ cells were grown in DMEM media supplemented with 10% fetal bovine serum (FBS), 100 U ml^−1^ penicillin and 100 μg ml^−1^ streptomycin at 37 °C in 5% CO_2_. Ovarian cancer PEO4/PEO1^44^ were cultivated in RPMI media supplemented with 10% fetal bovine serum (FBS), 100 U ml^−1^ penicillin and 100 μg ml^−1^ streptomycin and UWB1/UWB1 + BRCA1^46^ cells were grown in 50% RPMI media, 50% MEGM bullet kit (Lonza CC-3150) completed with 3% FBS, 100 U ml^−1^ penicillin and 100 μg ml^−1^ streptomycin at 37 °C in 5% CO_2_.

### Drug and reagents

The MRE11 inhibitor Mirin was from Sigma-Aldrich. The NSC-105808 DNA2 inhibitor was a gift from Gregorz Ira^24^. The DNA2 inhibitor was used at a concentration of 0.3 μM for 24 h. Hydroxyurea (Sigma-Aldrich) was dissolved in double-distilled H_2_O to obtain a 100 mM (7.6 mg ml^−1^) solution. Cisplatin (Sigma-Aldrich) was dissolved in PBS 10× to obtain a 5 mM stock. UV-C was used at 40 mJ cm^−2^ as described in the labeling scheme. The MUS81 vectors used for the genetic complementation experiments was a gift from by Ian Hickson^33^. All vectors were amplified in DH5α *Escherichia coli* and extracted with GeneJET Plasmid Midiprep Kit (ThermoFisher Scientific).

### RNA interference

All transient gene depletions were carried out using the Lipofectamine RNAiMax transfection reagent (Life Technologies), except for *EXO1* gene silencing that was performed using *Trans*IT-siQuest (Mirus). SMARTpool siRNA from Dharmacon were employed to deplete the following genes: *BRCA2* (L-003462-00, 10 nM, 24 or 48 h) as described^47^, *BRCA1* (L-003461-00, 50 nM, 48 h) as described^48^, *MRE11A* (L-009271-00, 40 nM, 48 h) as described^49^, *MUS81* (siRNA2: L-016143-01, 20 nM, 48 h) as described^50^, *POLD3* (L-026692-01, 50 nM, 48 h), as described^33^. The following genes were depleted with siRNA purchased from Ambion: *DNA2* (4390827, 10 nM, 48 h), *EXO1* (#1: 4392420 and #2: S17502, 40 nM, 48 h), *RAD51* (4390827, 50 nM, 48 h) and *MUS81* in rescue experiments (AM16708, 25 nM, 48 h). The *EME2* siRNA was purchased from Qiagen (GeneSolution 146956, 80 nM, 48 h). The *EME2* and *CtIP* siRNAs were custom made: *EME2* (5′-GCGAGCCAGUGGCAAGAGA-3′, 40 nM, 48 h) as described^32^ and *CtIP* (5′-GCUAAAACAGGAACGAAUC-3′, 50 nM, 48 h) as described^20^. Silencer select negative control siRNA (4390843, Ambion) was used for the control experiments.

### RT-qPCR and western blot analysis

The levels of siRNA-mediated knockdowns were determined by RT-qPCR or western blot. mRNAs was extracted with the PureLink RNA mini kit (Invitrogen) and cDNA was synthesized using the M-MLV reverse Transcriptase (Life Technologies) according to the manufacturer’s indications. RT-qPCR experiments were performed using the following primers: BRCA1 (5′- AGAAACCACCAAGGTCCAAAG-3′ and 5′-GGGCCCATAGCAACAGATTT-3′), BRCA2 (5′- AGGACTTGCCCCTTTCGTCTA-3′ and 5′-TGCAGCAATTAACATATGAGG-3′), CtIP (5′-AAGAGGAGGAATTGTCTACTGC-3′ and 5′-AGAATCTTGTCCCCTGTGGTGGA-3′), DNA2 (5′- ATTAGCATTTGGCGTGTGGC-3′ and 5′-CTTTCTGTGTTACCCCCGGT-3′), EME1 (5′-CTCATCCCTGAGGGCTAGAA-3′ and 5′-AGTTGAAAGAGTGGCGGGA-3′), EME2 (5′-AGGTGGAAGAGGCCCTGGTA-3′ and 5′-CCCTGCTGTGCAGAAGGAGA-3′), EXO1 (5′- CCTCGTGGCTCCCTATGAAG-3′ and 5′-AGGAGATCCGAGTCCTCTGTAA-3′), GAPDH (5′- GAGCCACATCGCTCAGAC-3′ and 5′-GACCAGGCGCCCAATAC-3′), MRE11 (5′- CCAGAGAAGCCTCTTGTACG-3′ and 5′-TTCCACCTCTTCGACCTCTTC-3′), MUS81 (5′- CTAACGAGAGGAGAGCCTGC-3′ and 5′-GAGTGGAGCCAAGGGAAAAGA-3′), and POLD3 (5′- GAGTTCGTCACGGACCAAAAC-3′ and 5′-GCCAGACACCAAGTAGGTAAC-3′). Reactions were realized using the iQTM SYBR® Green supermix (Bio-Rad) following to the manufacturer’s instructions. For each sample, normalization was performed using GAPDH. Results were expressed relative to indicated controls.

For western blot analysis, cells were lysed in a buffer containing 100 mM Tris-HCl, 4% SDS, 20% glycerol, β-mercaptoethanol (100 μl ml^−1^). 20 μg of protein extracts were loaded onto a NuPAGE™ Novex™ 3-8% Tris-Acetate Protein Gels, 1.0 mm, 15-well (ThermoFisher Scientific). Proteins were transferred onto a nitrocellulose membrane (GE HealthCare) for 1 h at 15 V using the dry transfer machine Pierce G2 (ThermoFisher Scientific) following the manufacturer’s instruction. Membranes were blocked for 1 h in TBS containing 0.1% Tween 20. Next, membranes were probed with the anti-RAD51 rabbit polyclonal (1:1000; 05-530-I Sigma-Aldrich), anti-EXO1 rabbit polyclonal (1:1000; provided by Dr. Zhongsheng You), anti-BRCA1 mouse monoclonal (1:1000; ab16781 Abcam), anti-BRCA2 mouse monoclonal (1:1000; OP95 EMD-Millipore), anti-β-actin HRP (1:5000; A3854 Sigma-Aldrich) or anti-β tubulin rabbit polyclonal (1:5000; sc-9104, Santa Cruz Biotechnology, Inc.) antibodies. Proteins were visualized using ECL (Pierce) according to the manufacturer’s instructions.

### DNA fiber assay

Briefly, asynchronously growing cells were labeled with two thymidine analogs: 20 μM 5-iodo-2′-deoxyuridine (IdU; Sigma-Aldrich) followed by 200 μM 5-chloro-2′-deoxyuridine (CldU; Sigma-Aldrich) for the indicated times^13, 51^. Cells were washed twice with PBS after the first pulse and treated with the indicated doses of the genotoxic agents. After the indicated times, cells were collected and resuspended in PBS at 100,000 cells per ml. A total of 2 μl of this cell solution was mixed with 8 μl of lysis buffer (200 mM Tris.HCl pH 7.5; 50 mM EDTA; 0.5 % SDS) on a glass slide. After 6 min, the slides were tilted at a 20–45° angle, and the resulting DNA spreads were air dried, fixed in 3:1 methanol/acetic acid and stored at 4 °C. The DNA fibers were denatured with 2.5 M HCl for 1 h, washed with PBS, and blocked with 5% BSA in PBS/ 0.1% Tween 20 for 1 h. DNA immunostaining was performed with rat anti-BrdU antibody (1:50; AbCys SA, ABC117 7513) for CldU and mouse anti-BrdU antibody (1:50; Becton Dickson, 347580) for IdU in a humid chamber at 37 °C for 1 h. The following secondary antibodies were used: anti-rat Alexa 488 (1:100; Molecular Probes, A21470) and anti-mouse Alexa 546 (1:100; Molecular Probes, A21123) at 37 °C for 45 min. The slides were air dried and mounted with Prolong Gold Antifade reagent (Invitrogen). Images were sequentially acquired (for double-label) with LAS AF software using TCS SP5 confocal microscope (Leica). A 63×/1.4 oil immersion objective was used. Images were captured at room temperature. The DNA tract lengths were measured using ImageJ and the pixel length values were converted into micrometers using the scale bars created by the microscope. *n* ≥ 150 fiber tracts scored for each data set. The statistics for all these experiments measuring changes in the size of the IdU or CldU tracts were calculated on the total number of DNA tracts measured in each given sample (usually *n* ≥ 250). For the fork restart experiments, the percentage of stalled forks was calculated on the basis of at least 250 tracts counted in each independent experiment. All DNA fiber experiments were performed in duplicate or triplicate, as indicated in the figure legends. Additional information on the minimal number of tracts that should be measured for a reliable estimation of changes in fork speed within a given sample can be found in refs. ^26, 52^.

### Electron microscopy

For the EM analysis of replication intermediates, 5–10 × 10^6^ U2OS or PEO1/4 cells were collected and genomic DNA was cross-linked by two rounds of incubation in 10 μg ml^−1^ 4,5′,8-trimethylpsoralen (Sigma-Aldrich) and 3 min of irradiation with 366 nm UV light on a precooled metal block^10, 26^. Cells were lysed and genomic DNA was isolated from the nuclei by proteinase K (Roche) digestion and phenol-chloroform extraction. DNA was purified by isopropanol precipitation, digested with PvuII HF in the proper buffer for 3–5 h at 37 °C and replication intermediates were enriched on a benzoylated naphthoylated DEAE–cellulose (Sigma-Aldrich) column. EM samples were prepared by spreading the DNA on carbon-coated grids in the presence of benzyl-dimethyl-alkylammonium chloride and visualized by platinum rotary shadowing. Images were acquired on a transmission electron microscope (JEOL 1200 EX) with side-mounted camera (AMTXR41 supported by AMT software v601) and analyzed with ImageJ (National Institutes of Health). EM analysis allows distinguishing duplex DNA—which is expected to appear as a 10 nm thick fiber, after the platimun/carbon coating step necessary for EM visualization—from ssDNA, which has a reduced thickness of 5–7 nm. The criteria used for the unequivocal assignment of reversed forks include the presence of a rhomboid structure at the junction itself in order to provide a clear indication that the junction is opened up and that the four-way junction structure is not simply the result of the occasional crossing of two DNA molecules^25^. In addition, the length of the two arms corresponding to the newly replicated duplex should be equal (b = c), whereas the length of the parental arm and the regressed arm can vary (a ≠ b = c ≠ d). Conversely, canonical Holliday junction structures will be characterized by arms of equal length two by two (a = b, c = d).

### Metaphase spreads

Cells were treated with 4 mM HU for 6 h, washed twice with PBS, and released for 24 h in fresh medium. During the last 4 h, 10 μM nocodazole was added to the medium. Cells were collected, washed and resuspended in 10 ml of warmed hypotonic solution (10 mM KCl, 10% FBS) for 10 min at 37 °C. Cells were fixed by adding 500 μl of cold fixation buffer (acetic acid 1: 3 ethanol). Cell pellets were washed four times with the cold fixation buffer and stored in this buffer at 4 °C overnight. The nuclei were spread on cold slides. The slides were air dried overnight and mounted with Prolong Gold Antifade (Invitrogen) with DAPI. Images were acquired with a fluorescence microscope (LEICA DMU 4000B; 63×/1.40-0.60 NA oil) coupled to the LEICA DFC345FX camera. The images were analyzed with ImageJ. At least 50 metaphases per sample were scored in each experiment.

### Neutral comet assay for DSB detection

A total of 700 cells were resuspended in 70 μl 0.5% low melting point agarose (Trevigen, 4250-050-02) and spread on a comet slide (Trevigen, 4250-200-03). Cells were lysed in a cold lysis solution (Trevigen, 4250-050-01) at 4 °C for 30 min. DNA migration was performed in TBE buffer at 1 V cm^−1^ for 30 min. Slides were washed in milliQ water, fixed with ethanol 70% for 30 min and dried at room temperature. Comets were labeled with SYBR^®^ Gold Nucleic Acid Gel Stain (ThermoFisher) for 30 min. Images were acquired with a fluorescence microscope (LEICA DMU 4000B; 20×/0.4 CORR) coupled to the LEICA DFC345FX camera. The images were analyzed using ImageJ. At least 150 comets were scored per sample in each experiment.

### Cell viability

Cell viability was determined using the Cell Proliferation Kit II (XTT, Roche)^53^. Briefly, cells were seeded at 13,000 cells per well in a 24-well plate the day prior to treatment. Cells were treated chronically with the indicated doses of HU and cell viability was assessed 3 days after. The absorbance was measured at 450 nm with a reference wavelength at 650 nm. Results were expressed as percentage of the untreated control.

### Immunofluorescence microscopy

After treatment with 4 mM HU, U2OS cells were extracted with 1× PBS containing 0.2% Triton X-100 and protease inhibitors (Pierce) for 5 min on ice prior to fixation with 3.2% paraformaldehyde. The cells were then washed extensively with IF wash buffer (1× PBS, 0.5% NP-40, and 0.02% NaN_3_), then blocked with IF Blocking Buffer (IF wash buffer plus 10% FBS) for at least 30 min. Anti-MUS81 mouse monoclonal (1:200; ab14387 Abcam) or anti-POLD3 mouse monoclonal (1:350; H00010714-M01 Abnova) antibodies were diluted in IF Blocking Buffer overnight at 4 °C. After staining with secondary antibodies (1:1000, conjugated with Alexa Fluor 594; A11032 Life Technologies) and Hoechst 33342 (1:1000, Sigma-Aldrich), samples were mounted using Prolong Gold mounting medium (Invitrogen). Epifluorescent microscopy was performed on an Olympus fluorescent microscope (BX-53) using a UPlanS-Apo 100×/1.4 oil immersion lens, Hamamatsu ORCA-Flash 4.0LT digital camera, and cell-Sens Dimension software. The raw images were exported into Adobe Photoshop, and for any adjustments in image contrast or brightness, the levels function was applied as previously described^54^. For foci quantitation, at least 150 cells were analyzed in triplicate.

### Statistical analysis

Statistical analysis was performed using Prism (GraphPad Software). The statistical significance in each case was calculated as indicated in each figure legend.

### Data availability

The authors declare that all relevant data supporting the findings of this study are available with the article and its Supplementary Information files, or from the corresponding author upon request.

## Electronic supplementary material

Supplementary Information
