# Supplementary material for: MRE11 and EXO1 nucleases degrade reversed forks and elicit MUS81-dependent fork rescue in BRCA2-deficient cells
Source: Nat Commun. 2017 Oct 16;8:860. doi: 10.1038/s41467-017-01180-5 (PMC5643552; doi:10.1038/s41467-017-01180-5)
Supplement: Supplementary file 1 — Supplementary Information [file 41467_2017_1180_MOESM1_ESM.pdf]

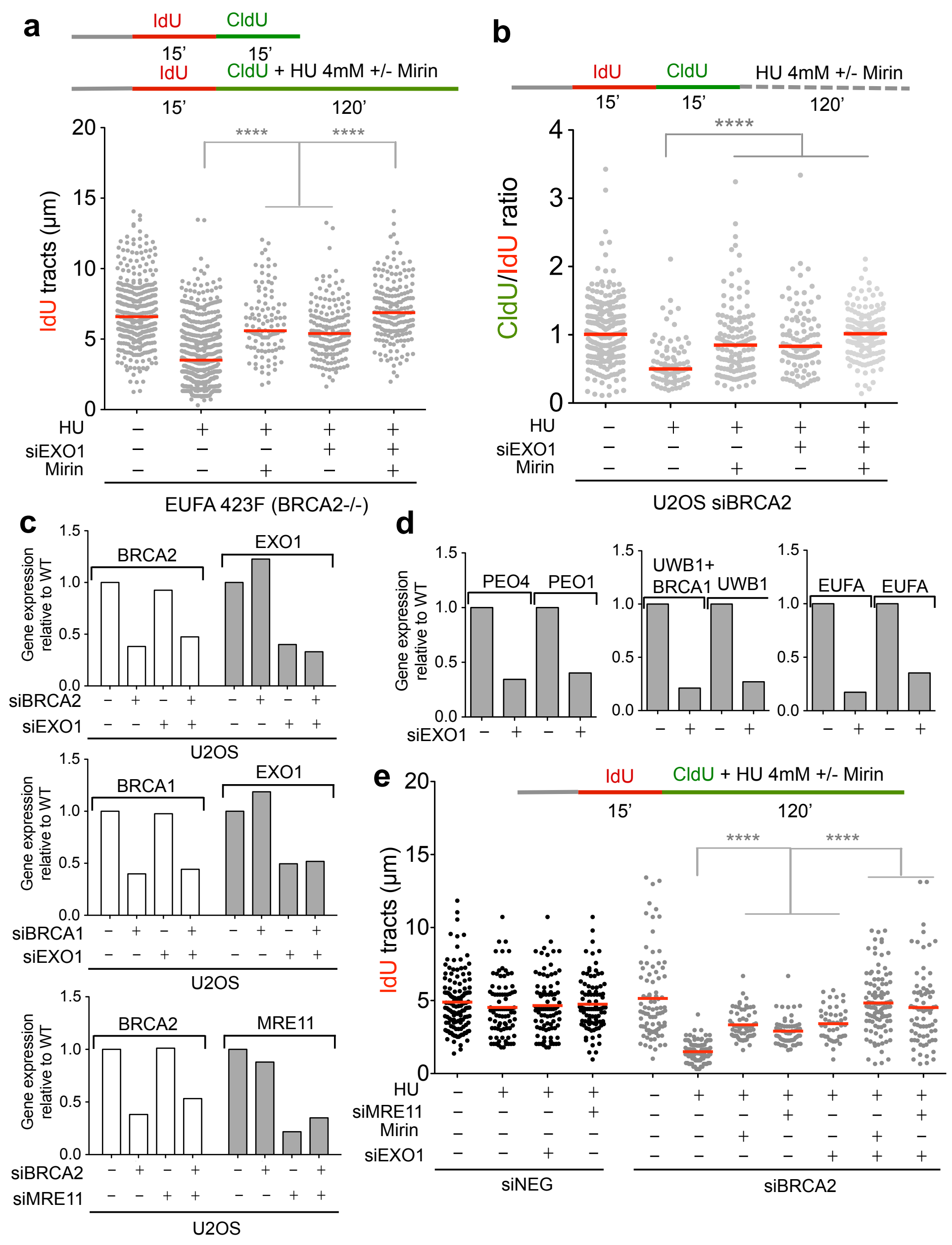

Supplementary Figure 1. MRE11 and EXO1 promote extended nascent strand degradation in HU-treated BRCA1- and BRCA2-deficient cancer cells. (a) Top, schematic of the single-molecule DNA fiber tract analysis. IdU, red; CldU, green. Bottom, size distribution of IdU tract length in BRCA2-deficient EUFA 423F cells in the presence and absence of HU. Cells were transfected with control siRNA or *EXO1* siRNA before IdU and CldU labeling. Mirin (50 μM) was added concomitantly with HU treatment. Out of 2 repeats; $n \geq 250$ tracts scored for each data set. Bars represent the median. Statistics: Mann–Whitney; ****, $P < 0.0001$. (b) Top, schematic of the single-molecule DNA fiber tract analysis. IdU, red; CldU, green. HU was added after CldU labeling. Bottom, size distribution of IdU tract length in BRCA2-depleted U2OS cells in the presence and absence of HU. Cells were transfected with control siRNA or *EXO1* siRNA before IdU and CldU labeling. Mirin (50 μM) was added concomitantly with HU treatment. Out of 2 repeats; $n \geq 250$ tracts scored for each data set. Bars represent the median. Statistics: Mann–Whitney; ****, $P < 0.0001$. (c) RT-qPCR analysis for *BRCA1*, *BRCA2*, *EXO1*, and *MRE11* gene deletions in U2OS cells. (d) RT-qPCR analysis for *EXO1* gene deletions in wild-type and *BRCA1*- or *BRCA2*-mutated cancer cells. (e) Size distribution of IdU tract length in BRCA2-deficient and proficient U2OS cells in the presence and absence of HU. Cells were transfected with control siRNA (siNEG), *EXO1* siRNA, or *MRE11* siRNA before IdU and CldU labeling. Mirin (50 μM) was added concomitantly with HU treatment. $n \geq 250$ tracts scored for each data set. Bars represent the median. Statistics: Mann–Whitney; ****, $P < 0.0001$.

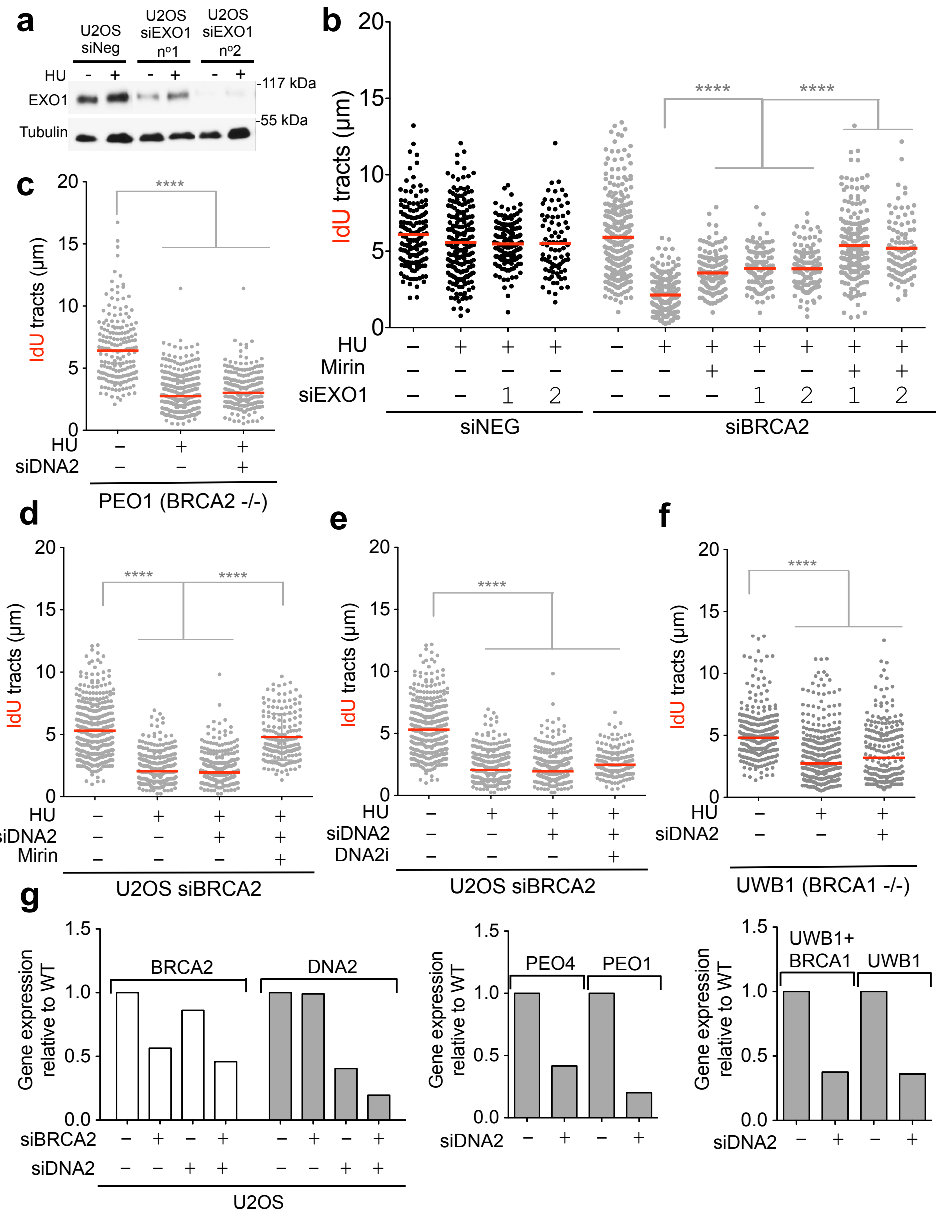

Supplementary Figure 2. DNA2 does not promote extended nascent strand degradation in HU-treated BRCA-deficient cancer cells. (a) EXO1 expression after knockdown with two different siRNA sequences. (b) Size distribution of IdU tract length in BRCA2-proficient and -deficient U2OS cells after *EXO1* knockdown with two different siRNA sequences in the presence and absence of HU. Cells were transfected with control siRNA (siNEG), *EXO1* siRNA1 or *EXO1* siRNA2 before IdU and CldU labeling. Mirin (50 μM) was added concomitantly with HU treatment. (c) Size distribution of IdU tract length in BRCA2-deficient PEO1 cells after *DNA2* knockdown in the presence and absence of HU. Cells were transfected with control siRNA or *DNA2* siRNA before IdU and CldU labeling. (d) Size distribution of IdU tract length in BRCA2-deficient U2OS cells after *DNA2* knockdown in the presence and absence of HU. Cells were transfected with control siRNA or *DNA2* siRNA before IdU and CldU labeling, as indicated. Mirin (50 μM) was added concomitantly with HU treatment. (e) Size distribution of IdU tract length in BRCA2-deficient U2OS cells after *DNA2* knockdown or treatment with the NSC-105808 DNA2 inhibitor in the presence and absence of HU. Cells were transfected with control siRNA, *DNA2* siRNA or the NSC-105808 DNA2 inhibitor before IdU and CldU labeling. (f) Size distribution of IdU tract length in BRCA1-deficient UWB1 cells after *DNA2* knockdown in the presence and absence of HU. Cells were transfected with control siRNA or *DNA2* siRNA before IdU and CldU labeling. For the experiments shown in panels b-f: Out of 3 repeats; $n \geq 250$ tracts scored for each data set. Bars represent the median. Statistics: Mann–Whitney; ****, P < 0.0001. (g) RT-qPCR analysis for *BRCA2* and *DNA2* gene deletions in U2OS, PEO1/4 and UWB1/UWB+BRCA1 cancer cells.

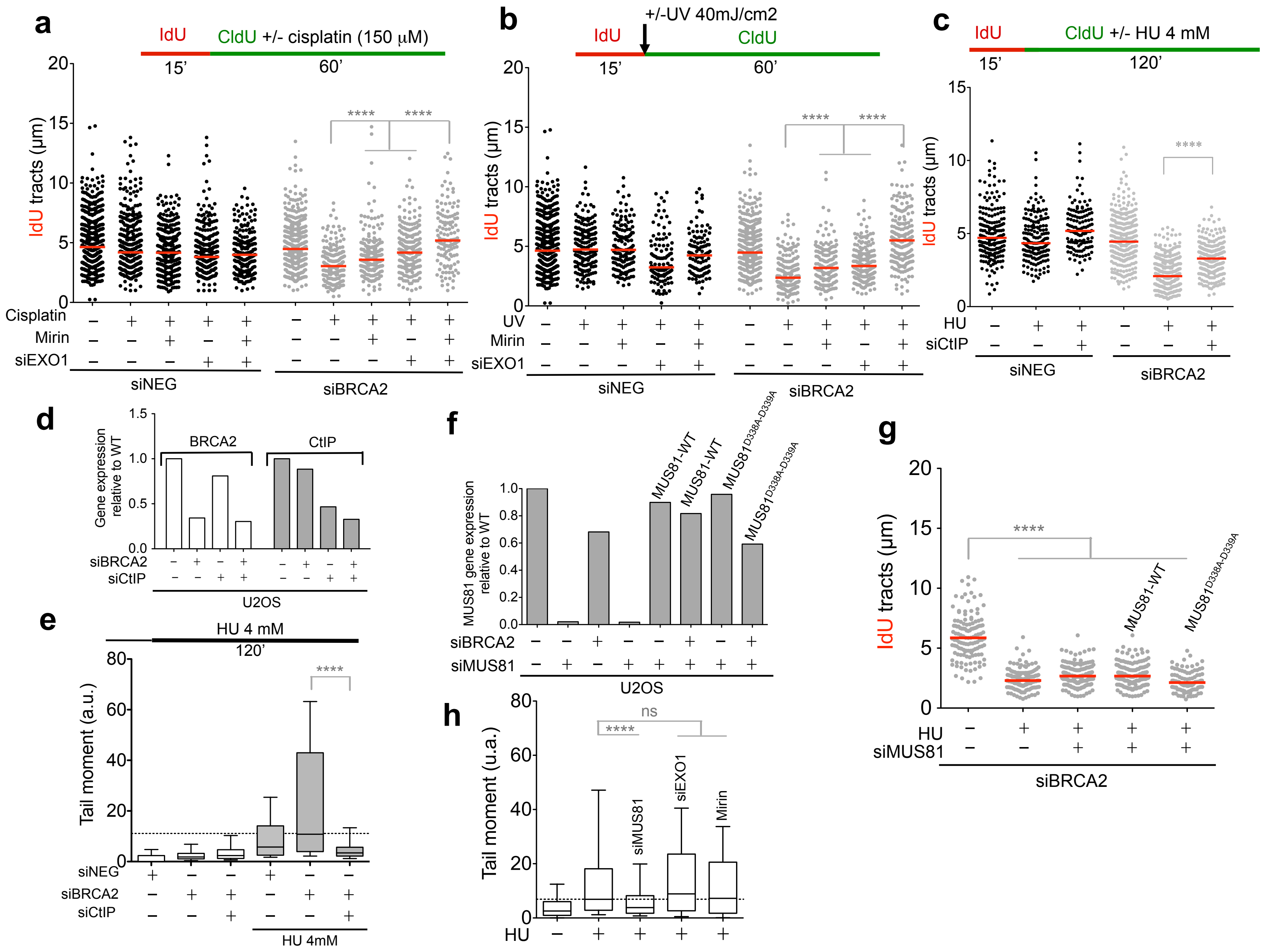

Supplementary Figure 3. Roles of MRE11, EXO1, CtIP, and MUS81 in fork degradation in BRCA2-deficent cells. (a) IdU tracts in BRCA2-deficient and proficient U2OS cells in the presence and absence of cisplatin. Cells were transfected with control siRNA (siNEG), *EXO1* siRNA, or *BRCA2* siRNA before IdU and CldU labeling. Mirin (50 μM) was added concomitantly with cisplatin treatment. Out of 2 repeats; $n \geq 250$ tracts scored for each data set. Bars represent the median. Statistics: Mann–Whitney; ****, $P < 0.0001$. (b) IdU tracts in BRCA2-deficient and -proficient U2OS cells in the presence and absence of UV. Cells were transfected with control siRNA (siNEG), *EXO1* siRNA, or *BRCA2* siRNA before IdU and

CldU labeling. Mirin (50 μM) was added concomitantly with CldU labeling. Out of 2 repeats; $n \geq 250$ tracts scored for each data set. Bars represent the median. Statistics: Mann–Whitney; ****, $P < 0.0001$. (c) IdU tracts in BRCA2-deficient and proficient U2OS cells upon CtIP depletion. Cells were transfected with control siRNA (siNEG) or *CtIP* siRNA before IdU and CldU labeling. Out of 2 repeats; $n \geq 250$ tracts scored for each data set. Bars represent the median. Statistics: Mann–Whitney; ****, $P < 0.0001$. (d) RT-qPCR analysis for *BRCA2* and *CtIP* gene deletions in U2OS cells. (e) Neutral Comet assay monitoring DSB formation upon CtIP depletion and treatment with 4 mM HU for 120 minutes. Cells were transfected with control siRNA, *BRCA2* siRNA, or *CtIP* siRNA. Out of 2 repeats; $n \geq 200$ comets scored for each data set. Whiskers the 10th and 90th percentiles. ****= $P < 0.0001$ (Mann-Whitney test). (f) RT-qPCR analysis for *MUS81* gene expression in U2OS cells. Cells were transfected with control *MUS81* siRNA, or *BRCA2* siRNA. MUS81 depleted cells were complemented with wild-type (MUS81-WT) or catalytically dead ($MUS81^{D338A-D339A}$) MUS81, when indicated. (g) IdU tracts in BRCA2-deficient U2OS cells with or without *MUS81* siRNA knockdown. MUS81 depleted cells were complemented with wild-type (MUS81-WT) or catalytically dead ($MUS81^{D338A-D339A}$) MUS81, when indicated. Cells were transfected with control siRNA, *MUS81* siRNA, or *BRCA2* siRNA before IdU and CldU labeling. Out of 3 repeats; $n \geq 250$ tracts scored for each data set. Bars represent the median. Statistics: Mann–Whitney; ****, $P < 0.0001$. (h) Neutral Comet assay monitoring DSB formation in BRCA2-proficient cells treated with 4 mM HU for 120 minutes. Cells were transfected with control siRNA, *MUS81* siRNA, or *EXO1* siRNA. Mirin (50 μM) was added concomitantly with HU treatment. Out of 2 repeats; $n \geq 200$ comets scored for each data set. Whiskers the 10th and 90th percentiles. ****= $P < 0.0001$ (Mann-Whitney test).

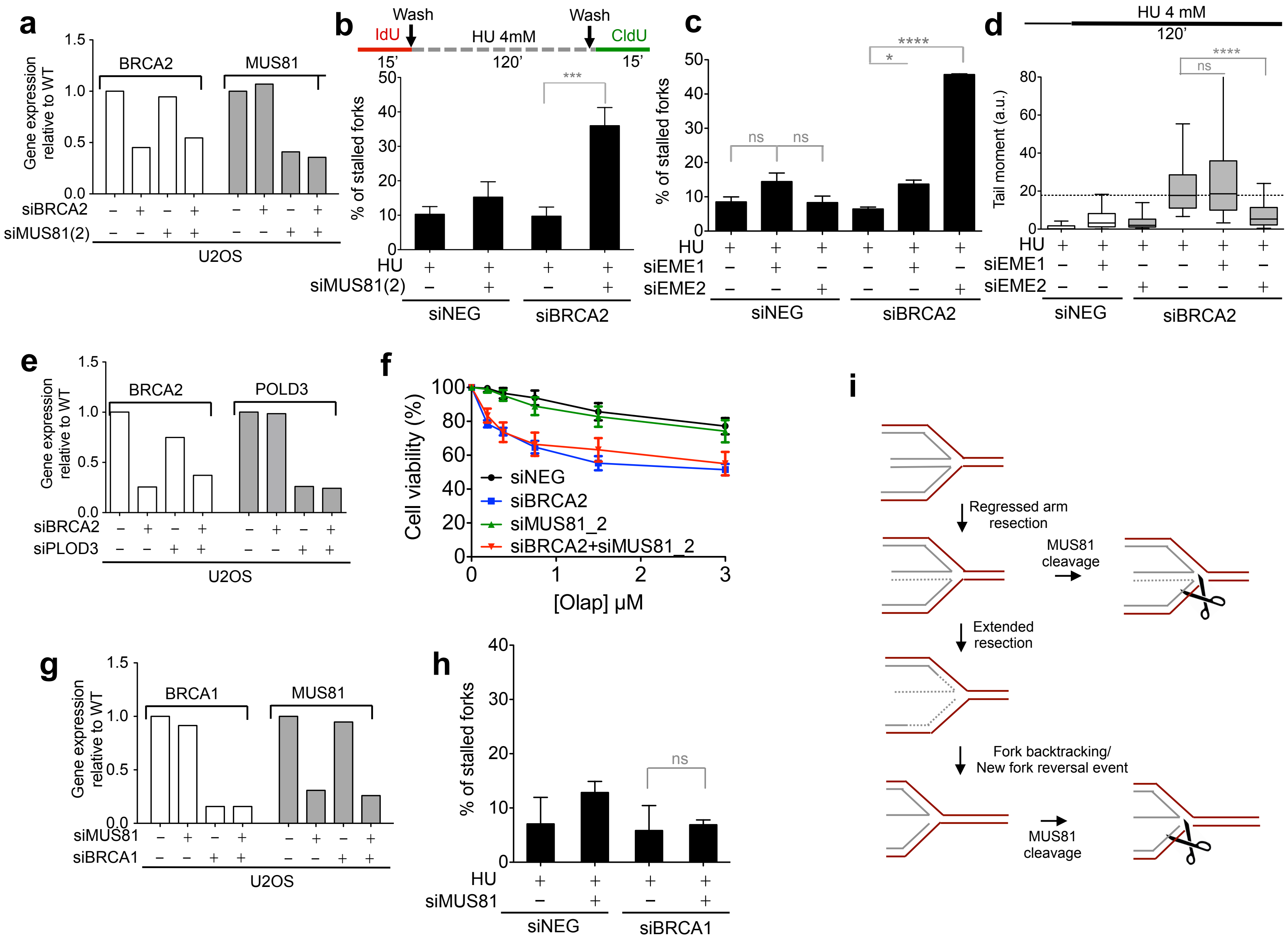

Supplementary Figure 4. MUS81 is required to rescue resected forks in BRCA2-deficient cells. (a) RT-qPCR analysis for *BRCA2* and *MUS81* gene deletions in U2OS cells using MUS81 siRNA2. (b) Quantification of restarting forks in BRCA2-deficient and -proficient U2OS cells with or without *MUS81* siRNA knockdown with MUS81 siRNA2. Out of 3 repeats, the percentage is established on at least 250 tracts scored for each data set. Mean shown. Errors, S.E.M. Statistics: unpaired t test; ***, $P < 0.001$. (c) Quantification of restarting forks in BRCA2-deficient and -proficient U2OS cells upon EME1 or EME2 depletion. Out of 3 repeats, the percentage is established on at least 250 tracts scored for each data set. Mean

shown. Errors, S.E.M. Statistics: unpaired t test; *, P < 0.05; ****, P < 0.0001. (d) Neutral Comet assay monitoring DSB formation upon EME1 or EME2 depletion and treatment with 4 mM HU for 120 minutes. Cells were transfected with control siRNA (siNEG), *BRCA2* siRNA, or *CtIP* siRNA. Out of 2 repeats; $n \geq 200$ comets scored for each data set Whiskers the 10th and 90th percentiles. ****= P <0.0001 (Mann-Whitney test). (e) RT-qPCR analysis for *BRCA2* and *POLD3* gene deletions in U2OS cells. (f) Cell viability assays 72 hours upon treatment with the indicated doses of PARP inhibitor (Olaparib). U2OS cells were transfected with control siRNA (siNEG), *BRCA2* siRNA, or two different *MUS81* siRNAs. Mean shown, $n = 3$. Errors, S.E.M. (g) RT-qPCR analysis for *BRCA1* and *MUS81* gene deletions in U2OS cells. (h) Quantification of restarting forks in BRCA1-deficient and -proficient U2OS cells with or without *MUS81* siRNA knockdown. Out of 2 repeats, the percentage is established on at least 250 tracts scored for each data set. Mean shown. Errors, S.E.M. Statistics: unpaired t test. (i) Schematic model for fork resection and MUS81 cleavage in BRCA2-deficient cells. The initial CtIP/MRE11/EXO1-mediated degradation of the regressed arm generates a reversed fork with a ssDNA tail that is cleaved by MUS81 to mediate fork restart. In the absence of MUS81 cleavage, the nucleolytic degradation might quickly proceed to degrade nascent strands behind the junction finally leading to extensively resected forks. After extended fork resection, reannealing of the parental strands might lead to "backtracking" of the fork. As a consequence of fork backtracking, a new reversal event may occur possibly promoting a new MUS81 cleavage event. This sequence of events would be effectively detected by fibers as fork backtracking, while EM would simply enrich for snapshots of the "slow steps" of a more extensive reaction (see also Vindigni and Lopes, Biophys. Chem 2016 for more details on the comparison of the results obtained by DNA fiber and EM).

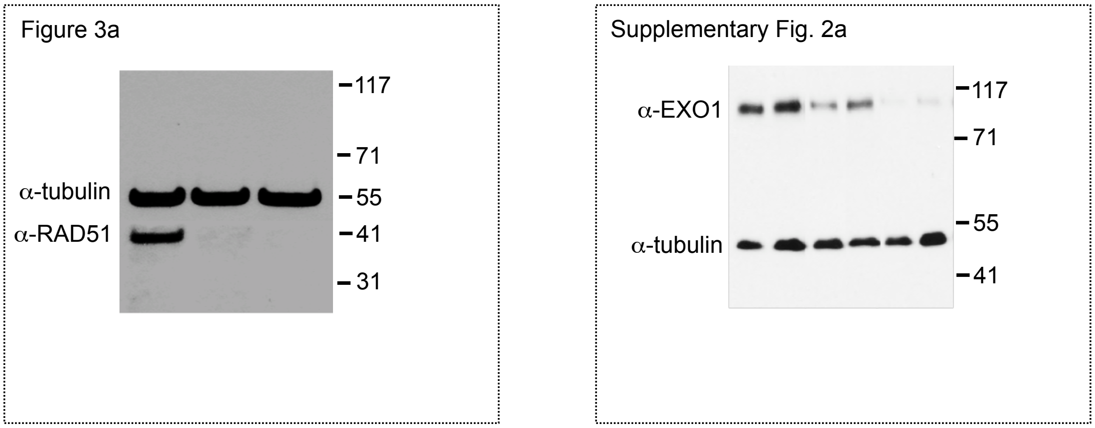

Supplementary Figure 5. Unprocessed films scans of immunoblots with corresponding size marker (kDa).
